# Are associations between home environment and preschool children’s sedentary time influenced by parental educational level in a cross-sectional survey?

**DOI:** 10.1186/s12939-020-01333-x

**Published:** 2021-01-09

**Authors:** Elviira Lehto, Reetta Lehto, Carola Ray, Riikka Pajulahti, Nina Sajaniemi, Maijaliisa Erkkola, Eva Roos

**Affiliations:** 1 Folkhälsan Research Center, Topeliuksenkatu 20, 00250 Helsinki, Finland; 2 Department of Education, Faculty of Educational Sciences, University of Helsinki, Helsinki, Finland; 3 Department of Food and Nutrition, University of Helsinki, Helsinki, Finland; 4 Department of Public Health, University of Helsinki, Helsinki, Finland

**Keywords:** Children, Sedentary time, Socioeconomic status, Home environment

## Abstract

**Background:**

Childhood obesity is a major public health concern, especially in low socioeconomic groups. Sedentary time (SED) is an important predictor of obesity. To be able to diminish SED it is important to find modifiable predictors of sedentary behavior. The home environment associated with children’s SED may vary by parental socioeconomic status. This study aims to clarify the association between parental educational level (PEL) and the home environment of 3–6-year-old children, and to examine how home environment associates with children’s SED, and whether PEL modifies these associations.

**Methods:**

A cross-sectional Increased health and wellbeing in preschools (DAGIS) study was conducted in 2015–2016 in Finland. The parents (*n* = 809) filled in questionnaires assessing PEL, and the home physical and social environment related to children’s SED. Children’s SED was measured with accelerometers, which the children (*n* = 745) wore for 1 week.

**Results:**

High PEL was associated with a home environment restraining sedentary behaviour compared with low PEL. Stricter descriptive norms about screen time, considering it important to limit the child’s screen time, and satisfaction about the child’s screen time associated with children’s lower SED. The association with parental psychological control and SED was influenced by PEL. In the PEL stratified analyses, however, the associations between psychological control and SED did not reach statistical significance.

**Conclusions:**

Future interventions aiming to decrease SED should pay attention to relevant factors in children’s sedentary behaviour home environment. It is important to acknowledge the possible PEL differences in these factors.

## Background

The prevention of unhealthy weight gain during childhood is a priority worldwide because of an increase in the prevalence of overweight and obesity among preschool-aged children [1]. Fortunately, many interventions that have concentrated on changing energy balance-related behaviours (EBRBs) among pre-schoolers and school children have shown a positive effect on the healthy weight status of children [2, 3]. Physical activity, sedentary behaviour, eating behaviour and occasionally sleep are components characterizing EBRBs [4], of which sedentary behaviour might be the one least studied. Sedentary behaviour refers to all waking activities expending less than 1.5 metabolic equivalents (METs) that are conducted in a sitting, reclining or lying posture [5]. Although sedentary behaviour is the opposite of physical activity when viewed from an energy expenditure angle, the two do not exclude each other, since the same child can both meet the physical activity recommendations of moderate-to-vigorous physical activity and have excessive sedentary time (SED) per day.

A Finnish study using accelerometers found that 5–6-year-olds spend half of their waking hours sedentary [6]. It is important to influence EBRBs, including sedentary behaviour, already at an early age, since EBRBs adopted during childhood track into adulthood [7]. A review by Jones et al. [8] identified moderate to large tracking of sedentary behaviours during the preschool age and from preschool into later childhood. This indicates the need to modify the associates of sedentary behaviour in children as early in life as possible.

The socioecological model stresses that the broader social and physical environments, including the home setting, are important determinants of children EBRBs [9]. The younger the child, the more central role the parents play in forming their sedentary behaviour by providing opportunities and by restricting them. For example, among 0–5-year-olds, screen-based SED was higher if their parents had more favourable descriptive norms or positive attitudes regarding screen time [10]. Higher SED was also noted among those preschool-aged children, whose parents promoted inactivity or screen time [11]. Higher parental psychological control over physically active play, on the contrary, accompanied with lower SED [11]. Among 6–11-year-olds, stricter rules concerning media use associated with less SED at home [12]. Moreover, a favourable social (i.e. rules on TV use) and physical (i.e. limited number of media devices in a child’s bedroom) family environment had an interactive association with lower SED [12]. These findings stress the importance that several components affecting children’s sedentary behaviour need to be considered simultaneously.

Certain differences in the home environment favouring sedentary behaviour have been attributed to parental education or income level. An Australian study [13] reported high-income families to have more equipment facilitating physical activity available to their school-aged children, although no differences were found in the educational level regarding the possession of such equipment. Nevertheless, relatively little research has been conducted among children under school age on socioeconomic status (SES) differences in the home environment that promote less sedentary behaviour [14–16].

According to Kremers et al. [4], social and physical home environment’s associations with EBRBs may vary between population subgroups and sociodemographic factors, including SES. This means that even though no SES differences in children’s SED have been found among Finnish preschool children [6, 17], SES may well influence the association between home environment and SED. Thus, not only the environmental factors might differ depending on SES, but also the association between factors in home environment and children’s SED may differ by SES. Only a few studies have examined this topic. A Finnish study [6] revealed that, although no SES differences in children’s SED was noted, the association between paternal and children’s SED differed according to SES. The SED of highly educated fathers associated with children’s lower SED, whereas among lower-educated fathers no association between paternal and children’s SED was found [6]. A study from the USA, however, did not find a moderating effect of maternal education / work status on the association between home environment and children’s sedentary time [18]. A study from the Netherlands showed stronger association between ethnic minority status and screen time among 4-year-old preschool children with higher maternal educational level, compared to those with lower maternal educational level [19]. In case the associations between environmental factors and children’s SED differ by SES, deeperknowledge about it could help to address relevant determinants among children with varying backgrounds.

The first objective of our present study was to examine whether modifiable home sedentary behaviour environments differ by parental educational level (PEL). With the term home sedentary behaviour environment, we refer to the social and physical factors at home that may affect children’s sedentary behaviours. Our second aim was to examine, how the home sedentary behaviour environment is associated with children’s objectively measured SED. Since we concentrate on home sedentary behaviour environment factors, we include in the SED measurement only SED outside the preschool hours. Third, we investigated whether the associations between home environment and children’s SED are influenced by PEL.

## Methods

### Participants

DAGIS (www.dagis.fi) is a study examining children’s EBRBs and aiming to diminish their socioeconomic differences. The cross-sectional data included in the present study were collected during autumn 2015 and spring 2016 in municipalities with socioeconomically diverse populations located in either southern or central Finland. We described the study design and recruitment process in more detail in Määttä et al. [20] and Lehto et al. [21], respectively. Eight of the eleven recruited municipalities agreed to participate in the study. We recruited 154 preschools in a randomised order, sixteen of which were later excluded due to unsuitability [21]. Of the eligible preschools, 86 (56%) agreed to participate in the study. All parents with 3–6-year-old children in these preschools were invited to participate in the study and received information letters with consent forms. Parents were asked to return the consent forms to the preschools if they agreed to allow their child to take part in the study. In total, 27% of the parents agreed to participate (*n* = 983). However, we excluded preschools (*n* = 20) in which less than 30% of the children participated in the study because of convenience reasons. This means that 90 parents who had given their written consent were excluded from the study. We received no data from 28 children due to them e.g. being ill, on vacation or otherwise absent during the study week, and these children were therefore excluded from the study. Overall, 66 preschools participated (43% of invited) in the study and we received data for 864 children (24% of invited).

### Measures

Questionnaires assessing the children’s EBRBs and home sedentary behaviour environments were sent home to the parents. Parents could choose whether they wanted to fill out a paper or electronic version of the questionnaire. Parents also kept a handwritten diary concerning their children’s SED. Research assistants placed an accelerometer around each child’s waist in the preschools. Both questionnaires and the accelerometers were returned to the preschools.

#### Sedentary time (SED)

SED was measured using Actigraph wGT3X-BT accelerometers (Actigraph, LLC, Pensacola, Florida, USA). Each child wore the accelerometers for 7 days, 24 h per day. In addition to the accelerometers, parents used a diary to track the hours their children had spent at preschool, possible hours spent not wearing the accelerometers and the times the child went to sleep in the evening and woke up in the morning. We used an epoch length of 15 s when downloading data from the accelerometers and set non-wearing time to 10 min or more consecutive zeros. To form the SED variable, we used cut-off points of 0–25 counts/15 s developed by Evenson et al. [22], since these have been reported to classify SED accurately in 5–15-year-old children [23].

A child’s SED variable was formed from two variables: SED during weekdays and SED during weekend days. Weekday SED was formed for out of preschool time with following criteria: The child had to be present at preschool for at least 2 days during the study week and had to attend for at least 360 min per day. Preschool hours were excluded from the total measurement time. Mean of at least two weekdays was calculated to form the weekday SED variable. The weekend SED variable was formed if the child had a dataset of at least 600 min per day for both weekend days. Both weekday and weekend SED were divided by the time that the child wore the accelerometer and multiplied by 60 to obtain the average SED per hour. We excluded data from the days when parents reported that their child was sick or absent from preschool. We also excluded night time sleeping hours but not any possible daytime nap times. The total SED variable was calculated with the following formula; (5 x mean SED during weekdays + 2 x mean SED during the weekend)/7. Valid accelerometer data in this variable existed for 745 children, of which two were defined as outliers (a distance of at least three standard deviations from mean) and removed. Thus, the final sample consists of 743 children.

#### Parental educational level (PEL)

We used PEL as an indicator of SES. The educational level of the parent, who filled out the survey questionnaire assessing the home sedentary behaviour environments, serves as an indicator of PEL. The parent who provided consent reported the highest educational level for both themselves and the other parent. The response categories were 1) comprehensive school, 2) vocational school, 3) high school, 4) bachelor’s degree or college, 5) master’s degree and 6) licentiate/doctorate. Answers were categorized as 1) low educational level (including categories 1–3), 2) middle educational level (category 4) and 3) high educational level (including categories 5 and 6). In the questionnaire, the respondent parent indicated whether they were the child’s mother, stepmother, father, stepfather or other guardian. None of the respondents was a stepmother or stepfather. Other guardians (*n* = 4) were excluded from the analyses. We then combined educational level information in the consent form with the parental status from the questionnaire and formed a variable indicating PEL.

#### Home sedentary behaviour environment

We conducted focus group interviews as part of the DAGIS study [24] and reviewed existing literature to help in the development of the home environment questionnaire for parents. We aimed to assess those aspects of the home environment that are proposed to associate with pre-schooler’s EBRBs. Questions adapted from international scientific literature describing previous studies [10, 11, 25–27] were translated into Finnish according to a translation and back-translation protocol. In addition, certain questions were modified to better suit the Finnish context. We included questions about following aspects of the social home environment: descriptive norm (parent’s opinion about suitable amount of screen time for 3–6-year-old children per day), number of screens accessible to the child, having rules limiting TV and other screen time, considering it important to limit the child’s screen time, parent’s own screen time (h/day) in the presence of the child (role modelling), and satisfaction with the child’s screen time. In addition, we used following constructs indicating home sedentary behaviour environment: psychological control, using screens as babysitter and promoting inactivity. Formed constructs and their items are presented in Table 1.

**Table 1:** Constructs indicating home sedentary behaviour environment, items included in the constructs, scale means for the items and Cronbach’s alphas of the constructs

Constructs indicating home sedentary behaviour environment	Items	Scale of the items	Mean (SD)	Cronbach’s α
**Psychological control**^a^	How often:	1–5 (never-always)		0.53
	Do you stop your child from playing actively for fear of him/her getting dirty?		1.53 (0.74)	
	Do you tell your child he/she will get hurt if he/she plays actively?		2.21 (0.86)	
	Do you discipline your child for playing too actively? (e.g. insisting on “time out”)?		1.56 (0.70)	
	Do you reward your child for being still?		1.51 (0.76)	
**Using screens as babysitters**^b^	My child uses electronic devices because:	1–5 (strongly disagree – strongly agree)		0.67
	It gives me the opportunity to get things done on my own.		3.46 (1.12)	
	It allows me to recover from a day at work/daily activities. (modified)		2.61 (1.28)	
	It focuses my child’s attention. (modified)		2.44 (1.24)	
**Promoting inactivity**^c^	How often do you:	1–5 (never-always)		0.28
	Carry your child if he/she does not want to walk?		1.97 (0.82)	
	Push your child in a stroller instead of allowing him/her to walk?		1.33 (0.67)	
	Drive your child when it is easy to walk?		2.29 (0.91)	

^a^ Construct formed according to O'Connor et al. [11], except one item dropped

^b^ Items adapted from Carson and Janssen [10] (1st and 3rd item originally from [28])

^c^ Construct formed according to O'Connor et al. [11]

#### Covariates

We included a child’s age and gender (girl/boy), parental status of the respondent (mother/father), and research time as covariates in the analyses because of the study design (e.g. seasonal differences in SED) and based on earlier literature concerning the possible covariates. Parents reported their child’s date of birth and gender in the questionnaire. Age was calculated by subtracting the date of the birth from the date of research and included as continuous variable in the analyses. Research time was divided into three categories, based on whether the participant filled out the questionnaires in September–October, November–December or January–April, and included as dummy variable in the analyses.

### Statistical analyses

Descriptive statistics such as means, standard deviations and percentages are reported according to PEL. Cronbach’s alphas were calculated and are presented in Table 1. We tested PEL differences of the home environment factors by means of chi-square tests and analyses of variance. The associations between home environment factors and children’s SED were examined with linear regression analyses (separate analyses for each independent variable). Thereafter, moderation analyses were conducted by including each independent variable at a time together with PEL as well as their interaction term in the models. These analyses were conducted with the PROCESS macro tool, version 3 [29], using bootstrapping at the level of 5000. The level for statistical significance was set at *p* < .05. We examined the data with the IBM statistical programme Statistics SPSS 23.0.

## Results

The sample characteristics are presented in Table 2. A total of 809 parents (94% of all participants) filled out the questionnaire assessing the home sedentary behaviour environment. Most respondents to the parental questionnaire were mothers, and nearly one-third had at least a master’s degree. The bulk of the children lived with both parents.

**Table 2:** Characteristics of the study population

	N	Percentage	Mean (SD)
**Children’s gender**	863		
Girls	413	48**%**	
Boys	450	52%	
**Children’s age**, years	864		4.7 (0.9)
**Children’s sedentary time**, minutes/hour	743		29.3 (4.5)
**Parental educational level**	792		
Low (high school, vocational school or less)	232	29%	
Medium (bachelor’s degree or equivalent)	327	41%	
High (master’s degree or higher)	233	29%	
**The child lives with**^a^	804		
Both parents	722	90%	
Only with mother	46	6%	
With mother and her new partner	15	2%	
Half time with mother and half time with father	19	2%	
Only with father	1	0.1%	
**Respondent of the parental questionnaire**^b^	802		
Mother	707	88%	
Father	95	12%	

*SD* Standard deviation

^a^One child living with other guardians not shown

^b^Four respondents, who were other guardians, were excluded from the analyses and are not shown

PEL differences in the home sedentary behaviour environment are presented in Tables 3 and 4. Psychological control was the only construct formed for environment that differed by PEL. High PEL was associated with parent’s lower levels of psychological control concerning children’s activities (Table 3). Children had access to an average of five screen devices in their home regardless of PEL (Table 4). Higher-educated parents more often believed that limiting their children’s screen time was important and more commonly considered that children’s screen time should not exceed one hour per day. Parents with a higher educational level spent less time at their screens in the presence of their children than their less-educated counterparts, during both weekdays and weekends.

**Table 3:** Constructs indicating home sedentary behaviour environment in total and by parental educational level (analysis of variance)

		Parental educational level			
	Total	Low	Medium	High	
**Construct,** range 1–5	**Mean (SD)**				***p*-value**
Psychological control (*n* = 786)	1.70 (0.5)	1.72 (0.5)	1.73 (0.5)	1.62 (0.4)	**0.03**
Using screens as babysitters (*n* = 793)	2.83 (0.9)	2.79 (1.0)	2.89 (1.0)	2.80 (0.9)	0.38
Promoting inactivity (*n* = 797)	1.86 (0.5)	1.91 (0.5)	1.82 (0.5)	1.87 (0.5)	0.09

*SD* Standard deviation

Statistically significant difference between parental educational level groups is in bold

**Table 4:** Home sedentary behaviour environment according to parental educational level (analysis of variance and chi-square test)

		Parental educational level			
	Total	Low	Middle	High	
	Mean (SD)/%				***p***-value
**Physical home sedentary behaviour environment**					
Number of screens in the household accessible to the child (*n* = 827)	5.00 (1.2)	4.96 (1.2)	5.01 (1.1)	5.03 (1.1)	0.80
**Social home sedentary behaviour environment**					
**Satisfaction**					
The parent is satisfied with the child’s screen time (agree somewhat or strongly)^a,b^ (*n* = 796)	69%	70%	69%	69%	0.98
**Importance**					
It is important for the parent to limit the child’s screen time (agree somewhat or strongly)^c^ (*n* = 795)	86%	82%	86%	91%	**0.01**
**Parent’s opinion about ‘suitable screen time’ (Descriptive norm for screen time)**					
Suitable screen time per day for 3–6-year-old children: a maximum of 1 h (*n* = 797)	42%	41%	38%	50%	**0.02**
**Role modelling for screen time**					
Parent’s screen time in the presence of the child (hour/day)^a^ (*n* = 797)	1.00 (0.7)	1.06 (0.9)	0.99 (0.7)	0.82 (0.6)	**0.001**
**Rules**					
Parent has rules limiting the child’s TV time (applies to families with a TV at home)^d^ (*n* = 776)	75%	73%	77%	76%	0.57
Parent has rules limiting child’s other screen time (applies to families with other screens besides TVs)^d^ (*n* = 781)	79%	80%	78%	79%	0.91

*SD* Standard deviation

Statistically significant differences between parental educational level groups are in bold

^a^ Statements adapted from Gonzalez-Gil et al. [25] (modified, except the item marked with ^b^)

^c^ Statement adapted from Lampard et al. [26] (modified)

^d^ Statements adapted from Pinard et al. [27]

Table 5 presents the results of linear regression analyses examining the associations between home sedentary behaviour environment and children’s SED. Stricter descriptive norm for screen time associated with a child’s lower objectively measured SED. A child’s SED was also lower, if the parent considered it important to limit the child’s screen time or was satisfied with a child’s screen time. Other home environmental factors did not associate with children’s SED, although the relations between children’s higher SED and parent’s own higher screen time in the presence of the child was near statistical significance (*p* = .059).

**Table 5:** Unstandardized linear regression coefficients and their 95% confidence intervals for the association between home sedentary behaviour environment and children’s sedentary time (SED) (*n* = 687–794)

	n	B (CI 95%)
Psychological control	794	−0.20 (− 0.80–0.40)
Promoting inactivity	705	− 0.21 (− 0.82–0.39)
Using screens as babysitter	701	0.13 (− 0.19–0.44)
Descriptive norm for children’s screen time	701	− 0.93^**^ (−1.53--0.34)
Number of screens in the household accessible to the child	707	0.02 (−0.24–0.29)
Parent has rules limiting the child’s TV time	687	−0.56 (−1.25–0.13)
Parent has rules limiting child’s other screen time	690	−0.11 (− 0.83–0.61)
Important for the parent to limit the child’s screen time	703	− 0.96^*^ (− 1.67- -0.08)
Parent’s screen time in the presence of the child (h/day)	705	0.39 (−0.02–0.80)
Parent is satisfied with the child’s screen time	704	−1.39^***^ (−2.01--0.76)

All analyses conducted separately for each independent variable and adjusted for children’s gender and age, parental status (mother/father), and season of conducting the study

Level of statistical significance ^*^p < .05, ^**^p < .01, p^***^ < .001

**Fig. 1:**
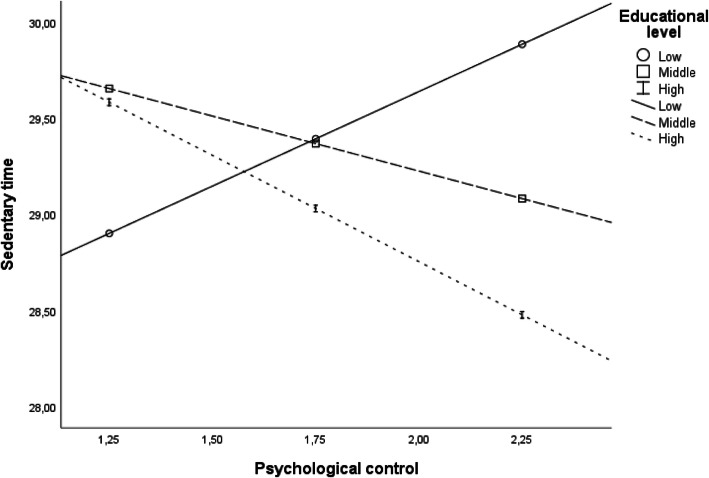
The associations between parental psychological control and children’s sedentary time (minutes/hour) presented separately by parental educational level groups

**Fig. 2:**
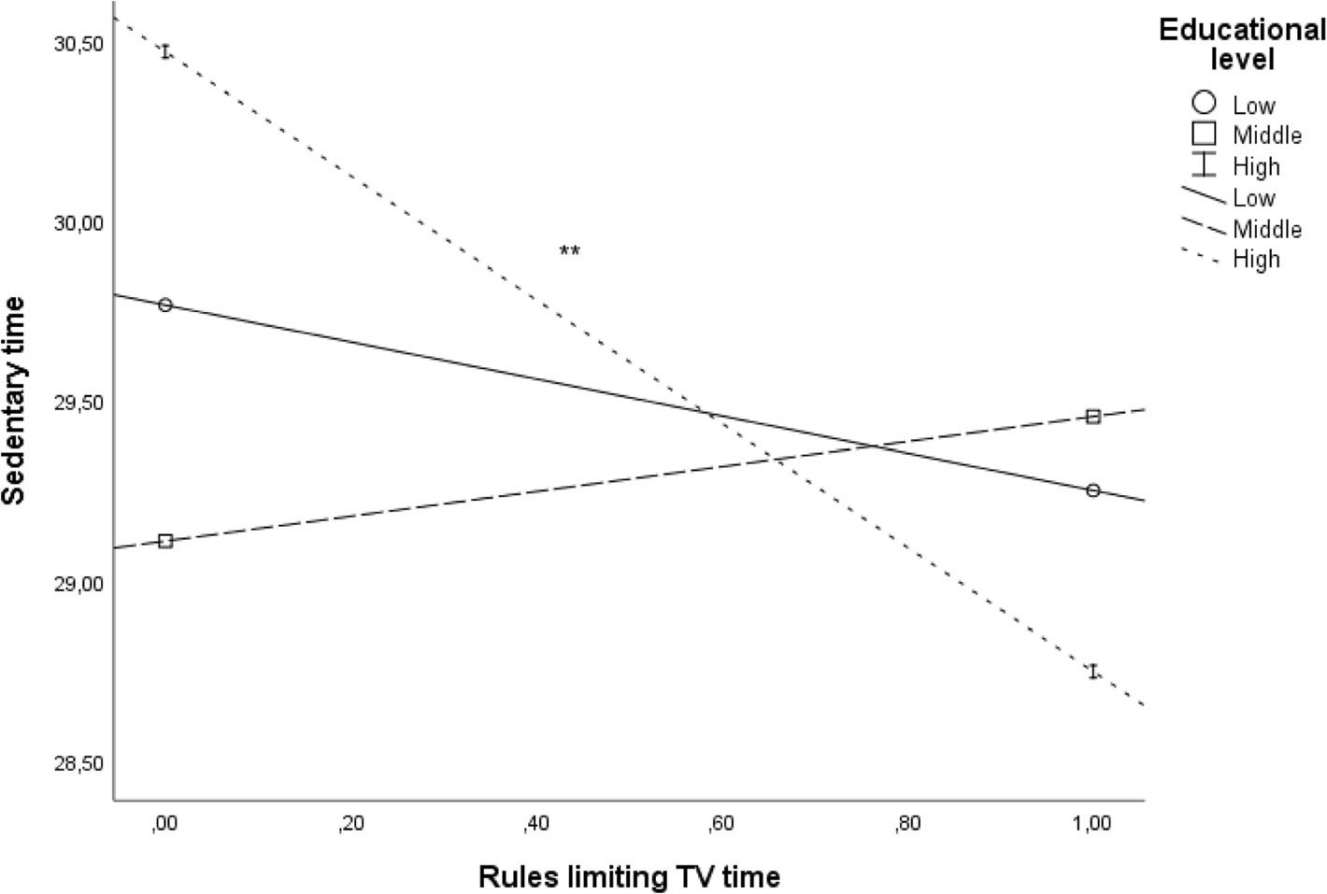
The associations between having rules limiting children’s TV time and children’s sedentary time (minutes/hour) presented separately by parental educational level groups (^**^p < .01)

In the association to a child’s SED, interactions existed for PEL and psychological control (*p* = .023). Interaction is presented in the Fig. 1. When separately analysing the associations between psychological control and children’s SED in different PEL groups, however, none of the associations was statistically significant: low PEL group B 0.98 (CI 95% -0.06-2.02), *p* = .07, middle PEL group B -0.62 (CI 95% -1.54-0.30), *p* = .19, high PEL group B -1.03 (CI 95% -2.30-0.24), *p* = .11 (data not shown). Interaction between PEL and having rules limiting TV viewing was near statistical significance (*p* = .053) (Fig. 2.). In PEL stratified analyses, having rules limiting children’s TV viewing was negatively associated with children’s SED only in the highest PEL group: low PEL group B -0.57 (CI 95% -1.83-0.68), *p* = .37, middle PEL group B 0.30 (CI 95% -0.82-1.42), *p* = .60, high PEL group B -1.78 (CI 95% -3.04--0.52), *p* = .006 (data not shown).

## Discussion

The present study examined PEL differences in home sedentary behaviour environment among 3–6-year-old children. Furthermore, we studied how home sedentary behaviour environment associates with children’s SED and whether these associations are influenced by PEL. High PEL was associated with a home environment promoting less sedentary behaviour compared with low PEL. Of all examined home environment factors, parents’ descriptive norms regarding children’s screen time, considering it important to limit a child’s screen time, and parental satisfaction about their child’s screen time were associated with children’s SED. Some of the associations with home environment factors and SED were influenced by PEL.

Several PEL differences in home sedentary behaviour environment among preschool-aged children were apparent. Higher-educated parents were more likely to emphasize importance of limiting children’s screen time, spent less time looking at screens when their children were present and expressed descriptive norms corresponding with official recommendations about the suitable amounts of screen time more often than less-educated parents. They also performed less psychological control to limit their children’s active playing compared to less-educated parents. Parents’ use of screen devices in the presence of their children was quite low, but we did observe PEL differences: the less-educated reported spending more time watching television or using other screens in their children’s presence, both on weekdays and during weekends. This supports the findings reported by Tandon et al. [30] in the Australian context that lower-SES parents watch more TV/DVDs with their under school-aged children than higher-SES parents do. Contrary to the previous studies examining SES differences in the home environment of school-aged children [13, 30], we found no differences in the number of accessible screen devices among pre-schoolers according to PEL.

In the present study, children’s higher SED associated with more favourable parental descriptive norm about screen time, a factor that has previously been associated with children’s higher screen time [10]. Children’s higher screen time has also been linked with higher number of screen devices in the household, whereas no association between the number of accessible screens and SED, measured with an accelerometer, was found [13] – a non-significant association confirmed in our study as well. These results indicate that although screen time and objectively measured SED might share some common associates, some distinctions can be made. In addition, some further social home environment factors can be considered potentially important for children’s SED. Lower SED was measured, for example, when parents perceived that limiting their child’s screen time is important. Even if not reaching statistical significance, parent’s own higher screen time in the presence of their child related with children’s higher SED (*p* = .059).

An essential aspect describing social home sedentary behaviour environment is having rules that limit children’s screen use. Therefore it is not surprising that higher SED has been reported among children whose parents have less such rules [30]. In our study, however, having rules limiting children’s TV time associated with children’s lower SED only in the high PEL group. Some further dissimilarities with other studies were also apparent. Higher SED has been documented among those children whose parents promote inactivity, whereas lower SED was more common among children whose parents pursued more psychological control [11]. We, on the contrary, did not find any relations between promoting inactivity or psychological control with children’s SED per se. The association of children’s SED and psychological control was, however, influenced by PEL. This means that although the association between psychological control and children’s SED was also non-significant in the PEL-stratified analyses, the direction or the magnitude of the associations differed by PEL: low PEL group showing a tendency towards positive association, and middle and high PEL groups towards a negative association. It is possible that although some factors are not yet related with SED among pre-schoolers, over time differences in the social and physical home environment play part in the formation of PEL differences in SED. Therefore, already child health centers and preschools could aim to communicate to parents that although psychological control might be based on understandable concerns on a child’s safety, especially low PEL parents should still encourage children to be physically active.

Very few studies have examined the moderating effect of parental SES on the association between home environment and sedentary behavior among young children. Østbye et al. [18] did not find a moderating effect of educational level on the association between home environment (accessibility of physical activity equipment, role modelling of physical activity, parental policies in support of physical activity) and SED. We examined other factors in the home environment, which may explain why PEL in our analyses influenced the associations of certain factors in the home environment with SED. As in the study by Østbye et al. [18], we did not find a moderating effect of PEL on the association between parental role modelling and children’s SED. This result contrasts a previous Finnish study [6] in which SED of highly educated fathers was associated with children’s lower SED, whereas that of lower-educated fathers showed no association. We can only hypothesize, whether the differences in the results are due to, for example, the younger age of the children in the present study: parental role modelling could be more important determinant for children’s SED among older children, then also enabling more variation based on PEL to be found. Since we used the PEL and role modelling information of the parent who answered the questionnaire, we were not able to examine role modelling of the mother or father separately.

### Strengths and limitations

The strength of the present study is the inclusion of a wide range of social and physical factors at home when examining their associations with preschool children’s sedentary behaviour and, moreover, PEL differences in these associations. Due to the extensive nature of the data collection for the DAGIS study, we had to ensure that participation in the study did not become too burdensome. Thus, we could only include a limited number of potential determinants of SED. The present study brings new insights on the linkage between home environment and SED, as well as on PEL differences in it, among an age group that has received only moderate attention, since previous studies have mainly concentrated on school-aged children. These findings also indicate that it might be necessary to pay attention to those factors that seem to be more relevant to SED in low-PEL families. Both child health centres and preschools could stress those factors in their communication with parents. Universally distributed information would not single anyone out, but could benefit the most those families, whose home environment is less favourable EBRB improving.

A major weakness of the present study is the low participation rate of the families (24%). We have no information of non-participants and cannot therefore correct for the low participation rate. This means that the results have to be interpreted with caution, since we cannot rule out the possibility of SES-dependent or less health-conscious non-participation, which would bias our results. Therefore, our results may present the EBRB-related home physical and social environments in a more positive light than in reality. The participants do not represent the whole Finnish population, but we have placed great emphasis in including participants with a variety of SES and other background factors by recruiting both urban and rural municipalities from different parts of the country and with relatively large SES differences within the municipalities. Although many of the questions assessing the home environment were adapted from previous studies in Western countries, they were not applicable in the Finnish context and had to be modified. The benefit of these adjustments is that the questions have been easier for the parents to answer, but comparing the results with other studies is not straightforward. This also applies to the constructs: we left out some items included in the original constructs [10, 11] due to their perceived irrelevance in Finnish context. This might have resulted in lower Cronbach’s alphas and therefore weakened the likelihood of establishing reliable results, especially for the construct indicating parental promotion of inactivity. Since the majority of the parents who filled in the questionnaire were mothers, it might be that our results are not generalizable to fathers. Future studies should, however, pay more attention in actively recruiting fathers to gain knowledge whether paternal and maternal impact on children’s SED differs. In addition, the impact of other sociodemographic factors such as marital or employment status could be important factors to pay attention to.

A previous study of the DAGIS survey showed no PEL differences in the SED of children [17]. This is rather surprising in light of our current study results, since the home environment for children with high PEL seems to be more favourable compared to low PEL families. Among the same children participating in the DAGIS study, screen time was higher among children with low PEL compared to high PEL [21]. Moreover, the impact of PEL on children’s screen time was mediated by descriptive norms for children’s screen time, parental screen use in the presence of their children, parental opinions on the importance of limiting children’s screen time and the societal pressures felt by parents for letting children use electronic devices [31]. One reason, why PEL differences existed in children’s screen time but not in SED, may be that the questions on home environment related to sedentary behaviour mainly focused on screen time and not on other sedentary behaviours among children such as drawing, listening to reading, etc. The absence of questions related to these other sedentary behaviours can be seen as a weakness of our study.

## Conclusions

High PEL was associated with a home environment restraining sedentary behaviour compared with low PEL. Few of the examined home environment factors related to sedentary behaviour and screen time were associated with children’s objectively measured SED. PEL acted as a moderator for certain factors associated with SED. Our results are only partly in accordance with studies conducted among school-children. These findings implicate that such instances as preschools and child health centers might want to deliver information on EBRBs which is adjusted more to meet the needs of low PEL groups. This could help less educated parents to modify the home environment to restrain sedentary behaviour from an early age.

## Data Availability

The data sets used during the current study are available from the corresponding author on reasonable request.

## Abbreviations

SES: Socioeconomic status
SED: Sedentary time
PEL: Parental educational level
EBRB: Energy balance-related behaviour
